# Improvement of halitosis by probiotic bacterium *Weissella cibaria* CMU: A randomized controlled trial

**DOI:** 10.3389/fmicb.2023.1108762

**Published:** 2023-01-17

**Authors:** Hee-seung Han, Haeji Yum, Young-Dan Cho, Sungtae Kim

**Affiliations:** Department of Periodontology, School of Dentistry and Dental Research Institute, Seoul National University and Seoul National University Dental Hospital, Seoul, Republic of Korea

**Keywords:** halitosis, probiotics, clinical study, safety, *Weissella cibaria*

## Abstract

Several *in vitro* and *in vivo* studies have evaluated the effect of probiotics on oral health; however, human clinical studies are still limited. Therefore, this study aimed to examine the effects of *Weissella cibaria* Chonnam Medical University (CMU)-containing tablets on halitosis. This randomized, double-blinded, placebo-controlled study included 100 adults with halitosis (age, 20–70 years). The participants were randomly assigned to the test group (*n* = 50) and control group (*n* = 50). One tablet [1 × 10^8^ colony forming units (CFU)/tablet] was to be taken each day over 8 weeks. The concentrations of volatile sulfur compounds (VSCs), bad breath improvement scores, and oral colonization of *W. cibaria* were measured. Psychosocial indicators including depression, self-esteem, oral health-related quality of life, and subjective oral health status were evaluated. Most variables were assessed at baseline, 4, and 8 weeks, and *W. cibaria* number and safety variables were assessed at baseline and 8 weeks. Intergroup comparisons were carried out using Student’s *t*-test, Chi-square test, or Fisher’s exact test on per-protocol analysis. Intragroup differences before and after intake were analyzed using the linear mixed-effect model (LMM). Per-protocol analysis was carried out in the test group (*n* = 45) and control group (*n* = 46). Total VSC was significantly lower in the probiotics group than in the placebo group at baseline (week 0, *p* = 0.046) and at 8 weeks (*p* = 0.017). The sum of hydrogen sulfide and methyl mercaptan did not differ significantly between the groups at baseline; however, it was significantly lower in the probiotics group than in the placebo group at week 8 (*p* = 0.012). Bad breath improvement (BBI) scores were significantly reduced at week 8 (*p* = 0.006) in the probiotics group. Statistically significant intergroup differences were observed for changes in the level of *W. cibaria* at week 8 (*p* < 0.001). Psychological indicators significantly improved from baseline to week 8 in the probiotics group. No safety issues were observed in either group. The levels of *W. cibaria* was higher in patients with halitosis using *W. cibaria* CMU-containing tablets. The subjective degree of bad breath and psychological indicators were improved in patients with halitosis using *W. cibaria* CMU-containing tablets.

## 1. Introduction

Halitosis refers to the bad oral breath during exhalation; it originates from the mouth, nasal cavity, upper respiratory tract, and upper digestive tract. It is generally a smell that makes others feel unpleasant (Scully and Felix, 2005). According to the recent international consensus (Seemann et al., 2014), halitosis is classified into genuine halitosis and pseudo-halitosis. Genuine halitosis is defined as an overt malodor, with an intensity exceeding socially acceptable levels, whereas pseudo-halitosis refers to a case that is not detected as malodor by the clinician wherein the patient claims to be suffering from halitosis despite unable to provide reliable evidence (Murata et al., 2002). Genuine halitosis is subdivided into intra-oral halitosis and extra-oral halitosis according to the cause (Seemann et al., 2014). The causes of bad breath in the oral cavity are poor oral hygiene, periodontal disease, coated tongue, food impaction in the interdental sites, unsanitary dentures, and inappropriate prostheses (Ortiz and Filippi, 2021). Bad breath is usually caused by volatile sulfur compounds (VSCs), namely hydrogen sulfide (H_2_S), methyl mercaptan (CH_3_SH), and dimethyl sulfide [(CH_3_)_2_S] (Tonzetich, 1977; Rosenberg, 1990). VSCs are mainly produced by the degradation of anaerobic bacteria in the oral cavity using proteins (L-cysteine, L-methionine containing sulfur) contained in gingival fissures, saliva, and food residues as substrates (Nakano et al., 2002a). *Treponema denticola*, *Prevotella intermedia*, *Prevotella loescheii*, *Porphyromonas endodontalis*, and *Porphyromonas gingivalis* produces significant amounts of H_2_S and CH_3_SH in human serum (Persson et al., 1990).

Many recent studies have reported alternative ways to eliminate bad breath without altering the normal flora, including the use of oral probiotics (Petti et al., 2008) or interventions involving *Lactobacillu*s (Keller et al., 2012; Teughels et al., 2013; Penala et al., 2016). Probiotics are believed to act through various mechanisms such as competitive inhibition of attachment and growth of pathogens, lowering of environmental pH levels, synthesis of antimicrobial substances, modulation of local and systemic immune responses, and direct antimicrobial effects (Fooks and Gibson, 2002). Specific strains of the genera *Lactobacillus, Streptococcus*, and *Weissella* are some of the most helpful probiotics used in the treatment or prevention of halitosis (Karbalaei et al., 2021). *Weissella cibaria* is a short-rod shaped gram-positive lactic acid bacterium (Bjorkroth et al., 2002). It is a dominant species in fermented foods, such as kimchi (Kwak et al., 2014). *W. cibaria* inhibits oral pathogens (Kang et al., 2006) and suppresses volatile VSCs (Lee et al., 2020). Kang et al. reported that *Fusobacterium nucleatum* could not produce VSCs in the presence of *W. cibaria* Chonnam Medical University (CMU) because the growth of *F. nucleatum* was inhibited by the hydrogen peroxide produced by *W. cibaria* (Kang et al., 2006).

Many *in vitro* and *in vivo* studies have evaluated the efficacy of probiotics in oral health; however, clinical studies on humans are still lacking. The objective of this study was to evaluate the impact of probiotic *W. cibaria* CMU-containing tablets on reducing the production of VSCs, bad breath improvement scores, and increasing *W. cibaria* oral colonization.

## 2. Materials and methods

### 2.1. Ethical considerations

This study was performed in accordance with the Declaration of Helsinki and Consolidated Standards of Reporting Trials (2010). Approval for the study was obtained from the Institutional Review Board of Seoul National University Dental Hospital (approval no. CRI19008) and registered in https://trialsearch.who.int (KCT0004291) on September 10, 2019. The participants were informed about the purpose and procedure of the study and that refusal to participate would not disadvantage them in any way. Written informed consent was obtained from all the participants prior to enrolment.

### 2.2. Study participants

According to a previous clinical research (Lee et al., 2020), the difference (mean ± standard deviation) in the improvement of bad breath between the test group and the control group was considered the primary outcome. The number of participants required for the independent t-test with significance level *α* = 0.05, bilateral test, power = 0.8, was 80. The initial sample size was planned as 40 in each group, considering a dropout rate of 20%, and 100 participants were enrolled in the current study. Using a computer-generated random list, random allocation sequences were generated for the placebo (control) and probiotics (test) groups. The participants were assigned to the placebo and probiotics groups in a 1:1 manner by block randomization, and we ensured that the male-to-female ratio was similar in both groups as much as possible.

The participants for this study were recruited from the Department of Periodontology, Seoul National University Dental Hospital. Participants were included if they: were able to comply with the protocol, were aged 20–70 years with >20 natural teeth, had no tongue impediment, such as glossitis or tongue cancer, had no severe periodontal disease when periodontal treatment, antibiotics, or tooth extraction are required during oral examination as soon as possible, and had total VSC concentration of 1.5 ng/10 ml or higher. Recruited patients should maintain routine oral hygiene, but periodontal treatment, oral hygiene treatment, and using oral products other than the provided toothbrush and toothpaste were prohibited. Exclusion criteria were as follows: presence of systemic diseases such as digestive disease, kidney disease, Sjogren’s syndrome, rheumatism, sinusitis or rhinitis, chronic gastritis, dry mouth, diabetes mellitus, uncontrolled hypertension (SBP 160 mmHg or DBP ≥ 100 mmHg), or allergies to lactose. Participants with compliance less than 80%, and individuals taking *Lactobacillus*-containing food, probiotic supplements, or medicine that could affect the outcomes within 1 week of their visit were dropped from the study. Those who received oral hygiene or periodontal treatment during the test period were also dropped out. Consumption of garlic, onions, green onions, and chives, which may affect bad breath, was prohibited the day before the visit. In addition, a smoking ban was provided from the start of the test to the end of the test. If the participants did not observe the above, the participants would be dropped out.

The assessments were conducted at Seoul National University Dental Hospital at baseline, 4, and 8 weeks. Participants and investigators were blinded to the intervention. In order to maintain double-blindness, envelopes containing tablets with a unique code of each assigned group were sealed and delivered to the research director. The allocations were not disclosed to the director until the end of the human application study except for cases of serious adverse events. Information related to the allocation of participants and distribution of tablets was managed by a third party who were not directly involved in the present study.

**Figure 1:**
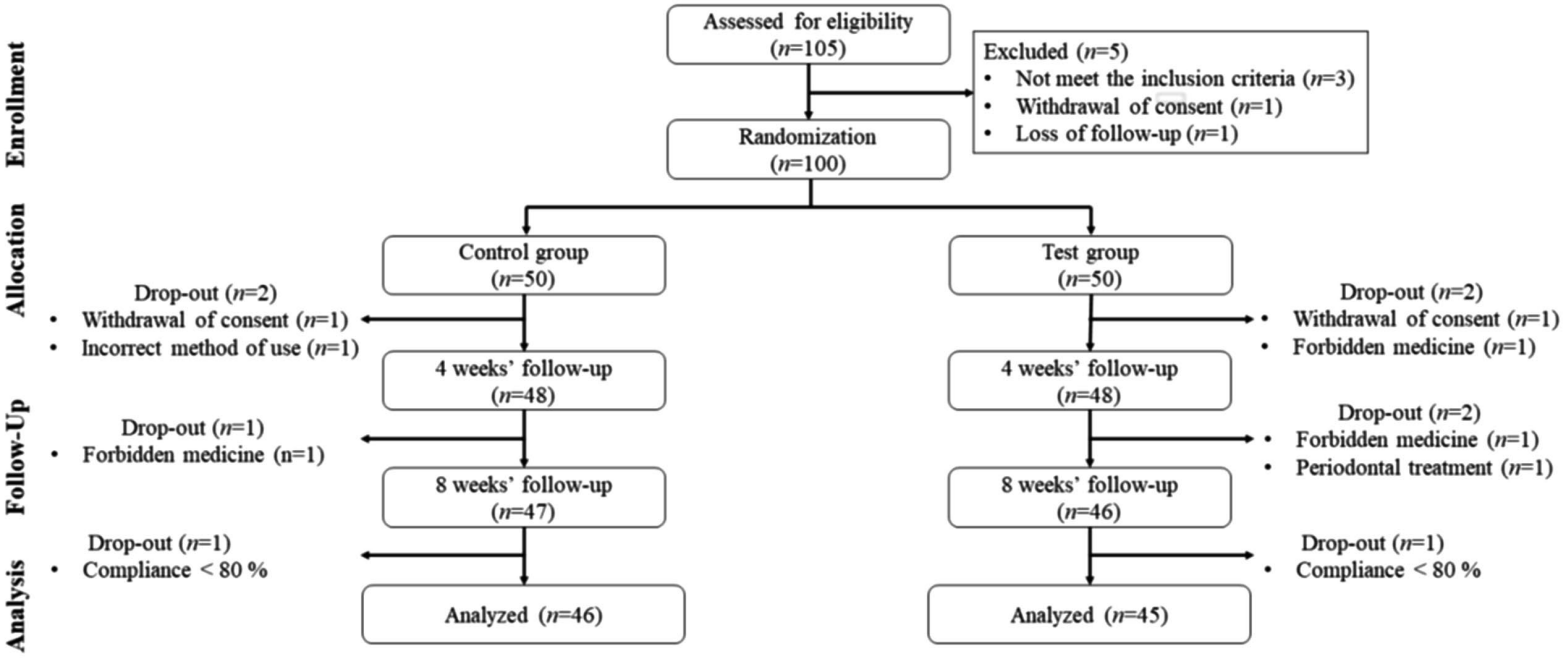
Flow chart of the study design.

A total of 105 participants were screened, 5 participants who did not meet the inclusion criteria or refused to participate during the 2-week run-in period were excluded. Therefore, 100 were randomly assigned to the placebo group (*n* = 50) or the probiotics group (*n* = 50). Nine additional participants were excluded from the 8-week intervention phase, and finally, 91 participants were included in the final analysis (Figure 1).

### 2.3. Study treatment

Each 800-mg probiotic tablet contained 1.0 × 10^8^ colony forming units (CFU)/tablet of *W. cibaria* CMU (oraCMU®; OraPharm Inc., Seoul, Republic of Korea). This strain is used as a food supplement in Korea. Other ingredients included isomalt, sucralose, peppermint-flavored powder, maltodextrin, magnesium stearate. The placebo was a tablet with a similar taste, texture, and appearance, but without *W. cibaria* CMU. It was obtained from the same manufacturer, and contained isomalt, sucralose, peppermint flavor, maltodextrin, and magnesium stearate. The participants were instructed to chew on one tablet every night before bedtime, after brushing their teeth. Participants were not allowed to consume water and food after the treatment. The intervention period lasted for 8 weeks.

### 2.4. Study design and protocol

This was a randomized, double-blind, placebo-controlled trial. The probiotic tablet was administered to the test group and a placebo tablet of the same shape was administered to the control group. The participants were interviewed about their dietary habits and oral health problems at two visits (4 and 8 weeks) to assess the compliance, potential side effects, dietary lifestyle survey, vital signs, whether taking forbidden drugs or health functional food or not, and measurement of halitosis. Participants were instructed to bring the remaining test food or control food at visit 3 (4 weeks) and visit 4 (8 weeks) after ingestion, and the remaining amount of test food or control food was reconfirmed. Compliance was calculated as follows.


$$\begin{array}{l} Compliance\\ =\frac{Number\;of\;test\;foods\;\left(or\;control\;foods\right)\;actually\;consumed}{Number\;of\;test\;foods\;\left(or\;control\;foods\right)to\;be\;consumed}\times 100 \end{array}$$

In principle, from the day the test food or control food was distributed to the day before this visit, the remaining food was returned to the researcher. Compliance was considered if the consumption rate was 80% or higher. All interventions took place in a double-blind manner; the participants were identified only by their registration number, and the intervention providers did not know who was in the test or control group. The entire study process lasted from September 06, 2019 to March 2020.

### 2.5. Measurement of halitosis

Halitosis was measured before intake and 4 and 8 weeks after intake using the concentration of VSCs and bad breath improvement (BBI) scores. The levels of VSCs were measured by Oral Chroma (CHM-2; FIS, Inc., Hyogo, Japan). The participants were instructed to refrain from talking for 3 min before the measurements and close the mouth for 30 s with a gastight syringe in the mouth. Thereafter, the examiner aspirated 1 ml of mouth air from the participant and injected it into Oral Chroma to measure the VSC concentration. The VSC analysis included the total VSC, which is sum of H_2_S, CH_3_SH, and dimethyl sulfide [(CH_3_)_2_S], and sum of hydrogen sulfide and methyl mercaptan. The concentration of VSC was measured on the morning upon following the guidelines for the study participants, including the instructions to refrain from eating after they had brushed their teeth in the evening until the next morning. The VSC analyzes were conducted according to a previous study (Kang et al., 2006). BBI scores were determined by self-estimation of the oral odor on a scale of 1–5 (Rosenberg et al., 1995): Score 1 - overall, symptoms improved significantly (very good), score 2 - overall, symptoms improved (excellent), score 3 - there is no difference from before intake (unchanged), score 4 - overall, symptoms worsened (exacerbated), and score 5 - overall, symptoms worsened significantly (extremely worse).

### 2.6. Quantitative analysis *Weissella cibaria*

The amount of *W. cibaria* was examined by quantitative PCR (qPCR). For this process, the middle part of the tongue was rubbed five times (Lee et al., 2020) with a cotton swab (iClean Swab; Biofact, Daejeon, Korea). Then, bacterial genomic DNA was extracted from the swab using a genomic DNA extraction kit (Biofact), according to the manufacturer’s instructions. Quantitative PCR (qPCR) was performed in a total volume of 10 μl using a Gene Probe PCR kit (Qiagen, Hilden, Germany), which contained 2 μl of the template, 200 nM of the primers, and 100 nM of the probe. The qPCR conditions were as follows: denaturation at 95°C for 3 min, followed by 45 cycles at 95°C for 3 s and 58°C for 10 s. qPCR was performed using a Rotor Gene Q system (Qiagen). The sequences of the primers and dual-labeled probe used for *W. cibaria* were as follows: forward, 5-GTGAAAGCCCTCAGCTCAAC-3; reverse, 5-CTACGCATTTCACCGCTACA-3 and 5-FAM-TGGAAACTGGATGACTTGAGTGCA-BHQ-3′. The number of bacterial cells per sample was calculated from a standard curve constructed using the diluted genomic DNA from *W. cibaria*.

### 2.7. Assessment of psychosocial health

A questionnaire on the depression, self-esteem, oral health-related quality of life, and subjective oral health status was administered to the participants to evaluate the social and psychological health indicators according to a previous study (Lee et al., 2021). Depression was assessed using the Center for Epidemiologic Studies Depression Scale (Radloff, 1977; Chon et al., 2001), which consisted of 20 items. The 20 questions are rated on a four-point Likert scale; higher the total score, greater the depression. Self-esteem was evaluated using an instrument developed by Rosenberg, which consists of 10 items (Rosenberg, 1965). Each item was rated on a five-point Likert scale, with a higher total score indicating greater self-esteem. Oral-health-related quality of life was measured using the shortened version of the Oral Health Impact Profile (Slade and Spencer, 1994), which consists of 14 items. Each item was rated on a five-point Likert scale, with a higher total score indicatinggreater oral-health-related quality of life. Subjective oral-health status was evaluated using an instrument (Park, 2010), which consists of 10 items. Each item is rated on a five-point Likert scale, with a higher total score indicating poor oral-health status.

### 2.8. Safety evaluation

The safety of this human clinical study was evaluated by monitoring the adverse events (AEs), vital signs, hematological findings (Hoffman and Monroe 3rd, 2001), indicators of liver function (Giovannini et al., 2008), kidney function, and electrolyte balance (Moreira et al., 2015) according to a previous study (Lee et al., 2020). AEs were monitored through interviews and self-report. In the event of an AE, based on the onset and disappearance of symptoms and signs as well as response actions were recorded, the event would be classified as either an AE or severe adverse event (SAE). In addition, blood samples were collected by nurses to determine the hematologic parameters and blood chemistry findings. All monitoring tests were performed at baseline and after 8 weeks.

### 2.9. Statistical analysis

A “per-protocol (PP)” analysis was performed on participants who completed the trial and whose compliance was ≥80%. Data with non-normal distribution were analyzed after being converted to ensure normal distribution. Intergroup comparisons of participant characteristics at baseline were carried out by Student’s *t*-test for continuous variables and Chi-square test or Fisher’s exact test for categorical variables. The compliance rates of the groups were compared using the Student’s *t*-test. Intergroup comparisons of improvements in bad breath at 4 and 8 weeks were carried out using Student’s *t*-test. Intergroup differences according to the intake period and intragroup differences before and after intake were analyzed using the linear mixed-effect model (LMM), with group, time, and interaction between group and time (group*week) included as random and fixed effects. Variables with significant differences between the groups (total VSC concentration and alcohol consumption) were corrected. The microbial index was analyzed by converting it to a log_10_ DNA copy value. For vital signs and hematological findings, differences between groups according to the intake period and differences within groups before and after intake were analyzed using the LMM. Adverse reactions were described in terms of the number of occurrences, type, symptom severity, and reaction to the test food by group and were compared between groups using the chi-square test or Fisher’s exact test. Statistical analysis was performed using SAS version 9.4, and a two-sided test was performed, and results with a *value of p* ≤0.05 was considered significant.

## 3. Results

### 3.1. Study population

A total of 100 out of 105 screened patients were recruited and included in the study. Fifty patients each were allocated to either the control or test group. The test group (probiotics, *n* = 45) and control group (placebo, *n* = 46) underwent a per-protocol analysis. A detailed description on patient recruitment is presented in Table 1.

**Table 1:** Baseline characteristics of the subject in the placebo and probiotic groups.

Variables	Placebo	Probiotic	Value of *p*^†^
Age (year)	51.3 ± 1.3	52.1 ± 1.2	0.655
Gender (male/female)	15/31	13/32	0.701
Body weight (kg)	63.2 ± 1.7	63.5 ± 1.7	0.898
Total volatile sulfur compound (ng/10 ml)	15.1 ± 3.1	8.2 ± 1.4	0.046
Alcohol drinker (yes/no)	21/25	12/33	0.060
Alcohol amount (abstention/less than 1 bottle/1–3 bottles/more than 4 bottles, per week)	25/17/4/0	33/7/3/2	0.043
Smoker (yes/no)	3/43	0/45	0.242
Smoking amount (cigarettes/day)	0.8 ± 0.5	0.0 ± 0.0	0.128
Compliance (week 4)	96.1 ± 0.8	96.8 ± 0.8	0.552
Compliance (week 8)	96.8 ± 0.8	97.7 ± 0.8	0.432

Values are mean ± SE (all such values). ^†^Student’s *t*-test for continuous variables and Chi-square or Fisher’s exact test for categorical variables were used to compare the difference between the groups.

No significant differences were found in any of parameters except total VSC (*p* = 0.046) and alcohol consumption (*p* = 0.043) between the probiotics and placebo groups. Total compliance for 8 weeks was 96% or higher in both groups, with no significant difference.

### 3.2. Measurement of halitosis

**Figure 2:**
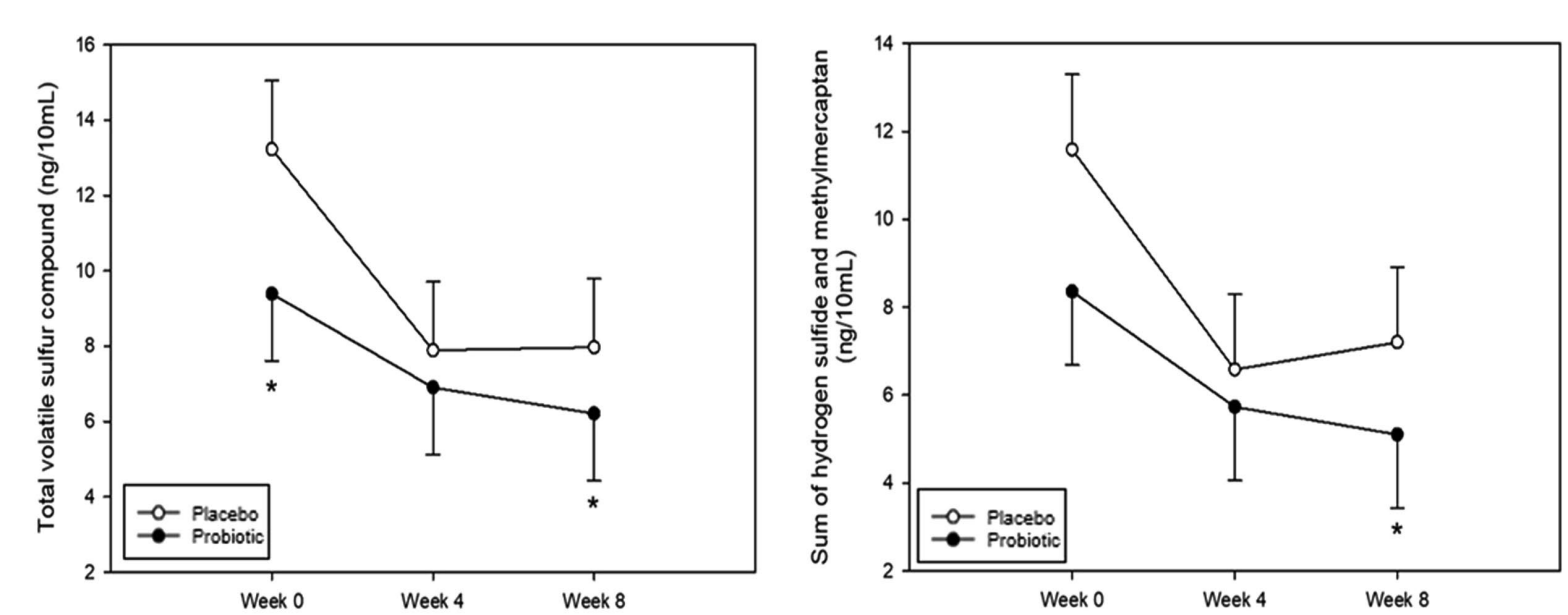
Analysis of volatile sulfur compounds. **(A)** Total volatile sulfur compound (VSC). Total VSC was significantly lower in the probiotics group than in the placebo group at baseline (week 0, *p* = 0.046) and at week 8 (*p* = 0.017). **(B)** Sum of hydrogen sulfide (H_2_S) and methyl mercaptan (CH_3_SH). The sum of H_2_S and CH_3_SH levels was significantly lower in the probiotic group than in the placebo group at week 8 (*p* = 0.012). Each line represents the least squares mean (LSmean) ± standard error (SE). *Value of *p* ≤ 0.05, Student’s *t*-test.

**Figure 3:**
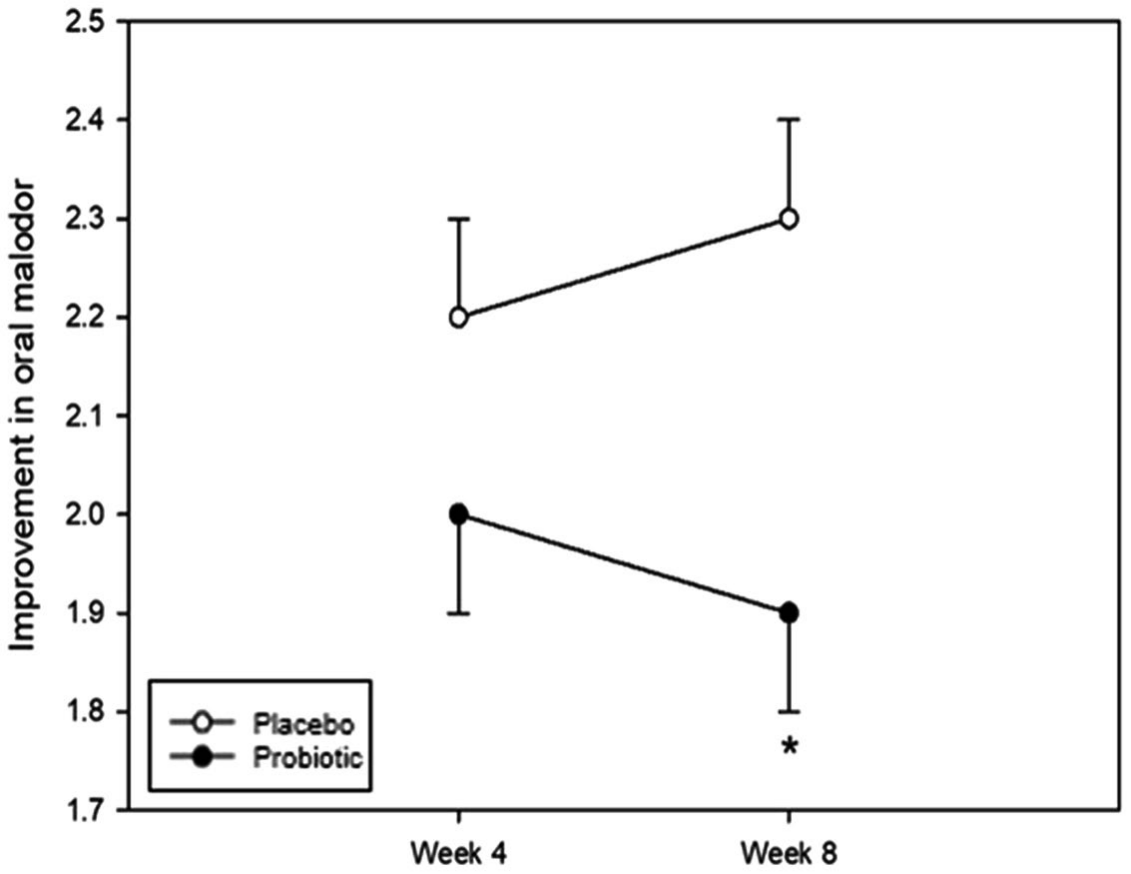
Analysis of bad breath improvement (BBI) scores. The BBI score was not significantly different between the groups at week 4; however, there was a significant difference at week 8 (*p* = 0.006). Each line represents the LSmean ± SE. *Value of *p* ≤ 0.05, Student’s *t*-test.

Halitosis was measured using the total VSC, sum of H_2_S and CH_3_SH levels, and BBI score. The measurements of the total VSC and the sum of H_2_S and CH_3_SH are presented in Figure 2 and Table 2. Total VSC was significantly lower in the probiotics group compared to the placebo group at baseline (week 0, *p* = 0.046) and at 8 weeks (*p* = 0.017). As shown in Table 2, the sum of H_2_S and CH_3_SH did not differ significantly between the groups at baseline; however, it was significantly lower in the probiotics group than in the placebo group at week 8 (*p* = 0.012). Analysis of the group differences according to the intake period with LMM showed no significant differences in any of the indicators. The BBI scores are presented in Figure 3 and Supplementary Table S1. There was no significant difference between the groups in the mean BBI scores at week 4 and changes in BBI scores from week 0 to week 4, but there was a significant difference at week 8 (*p =* 0.006). The placebo group showed an increase in the BBI scores, and the probiotics group showed a decrease in the BBI scores.

**Table 2:** Total volatile sulfur compound and sum of H_2_S and CH_3_SH at 0(baseline), 4, and 8 weeks (unit: ng/10 ml).

Variables	Placebo	Probiotic	Value of *p*^†^	Value of *p*^‡^
Total VSCs				
Week 0	13.23 ± 1.82	9.39 ± 1.78	0.046	
Week 4	7.89 ± 1.82	6.90 ± 1.78	0.130	
Week 8	7.97 ± 1.82	6.21 ± 1.78	0.017	0.642
value of *p*^§^	0.018	0.153		
Sum of hydrogen sulfide and methyl mercaptan				
Week 0	11.59 ± 1.71	8.36 ± 1.68	0.057	
Week 4	6.58 ± 1.71	5.73 ± 1.68	0.156	
Week 8	7.20 ± 1.71	5.10 ± 1.68	0.012	0.703
value of *p*^§^	0.029	0.108		

Values are LSmean ± SE (all such values). ^†^Student’s *t*-test was used to compare differences between the groups. ^‡^Linear mixed-effect model adjusted with total volatile sulfur compound, alcohol drinker and alcohol amount at baseline was used to analyze the effects of group* week. ^§^Linear mixed-effect model adjusted with total volatile sulfur compound, alcohol drinker and alcohol amount at baseline was used to analyze the difference within each group.

### 3.3. Quantitative analysis of *Weissella cibaria*

**Figure 4:**
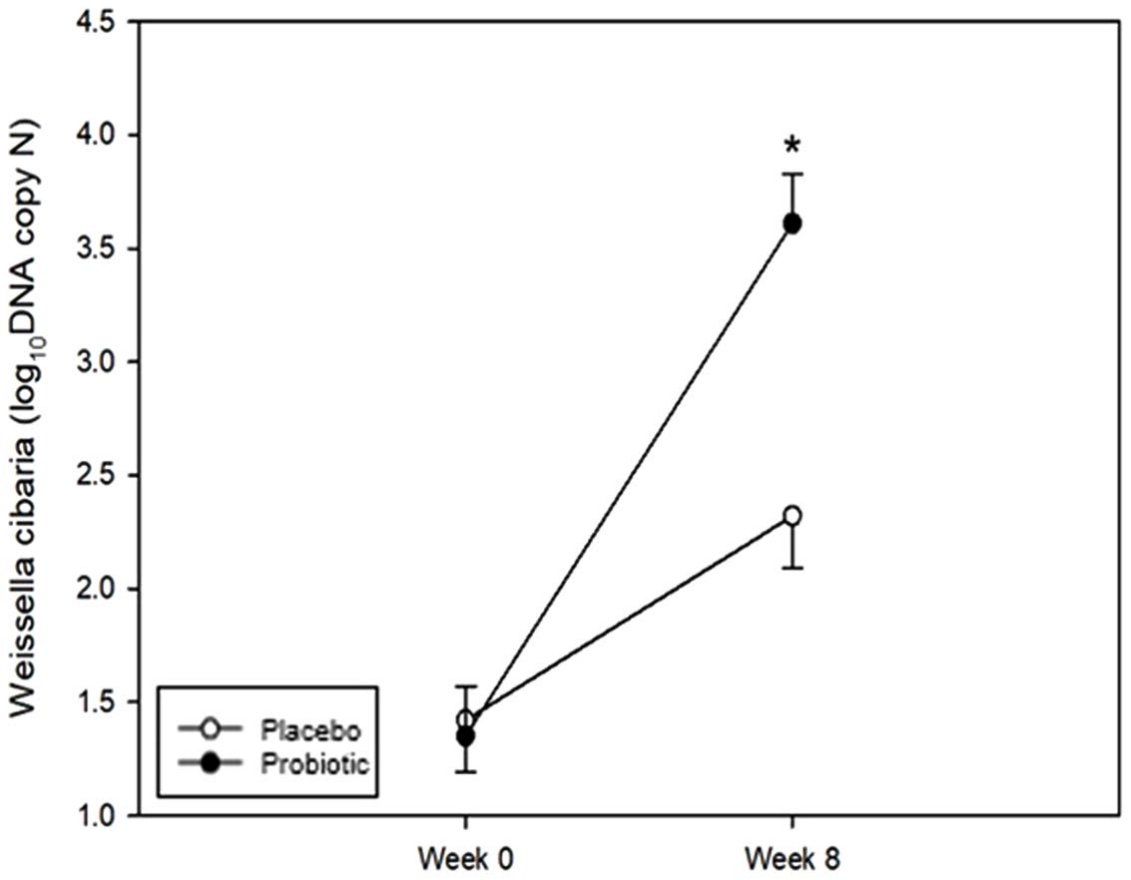
Changes in *Weissella cibaria.* The levels of *W. cibaria* were not significantly different between the groups at week 0 (*p* = 0.619). The levels of *W. cibaria* were significantly higher in the probiotic group than in the placebo group at week 8 (*p* < 0.001). Each line represents the LSmean ± SE. *Value of *p* ≤ 0.05, Student’s *t*-test.

Quantitative values detected in the tongue at each time point were analyzed to confirm the oral colonization of *W. cibaria*. Quantitative values were analyzed by conversion to log_10_ DNA copy values (Figure 4). Table 3 shows the results of the comparative analysis and statistical significance between the test and placebo groups. No statistically significant differences in the levels of *W. cibaria* were found between the groups at week 0 (*p* = 0.619). However, at week 8, the levels of *W. cibaria* were significantly higher in the probiotics group than in the placebo group (*p* < 0.001). There was a significant difference between the groups in terms of the changes in the proportion *W. cibaria* from baseline to 8 weeks (*p* < 0.001).

**Table 3:** Number of *Weissella cibaria* measured at 0 and 8 weeks (unit: log_10_DNA copy N).

Week	Placebo	Probiotic	Value of *p*^†^	Value of *p*^‡^
Week 0	1.42 ± 0.23	1.35 ± 0.22	0.619	
Week 8	2.32 ± 0.23	3.61 ± 0.22	<0.001	<0.001
Value of *p*^§^	<0.001	<0.001		

Values are LSmean ± SE (all such values). ^†^Student’s *t*-test was used to compare differences between the groups. ^‡^Linear mixed-effect model adjusted with total volatile sulfur compound, alcohol drinker and alcohol amount at baseline was used to analyze the effects of group*week. ^§^Linear mixed-effect model adjusted with total volatile sulfur compound, alcohol drinker and alcohol amount at baseline was used to analyze the difference within each group.

### 3.4. Evaluation of psychosocial health

As shown in Supplementary Table S2, depression (*p* = 0.049), oral health-related quality of life (*p* = 0.001), and subjective oral health status (*p* = 0.007) significantly improved from baseline to week 8 in the probiotics group. However, there were no significant differences between the groups at each visit (Student’s *t*-test) and according to the intake period (LMM) for all indicators.

### 3.5. Safety evaluation

Vital signs, hematological findings, blood chemistry results, and all AEs were monitored to evaluate the safety of this clinical study (Supplementary Tables S3–S6). During the study period, some of these safety variables showed significant intragroup differences; however, there were no clinically significant changes in any of the indicators. In addition, no SAEs occurred, the symptoms of the AEs were mild, and no reaction to the test food was reported (Supplementary Table S6). There were no significant differences between the groups in terms of the occurrence, type, symptom severity, or reaction to the test food.

## 4. Discussion

This study aimed to evaluate the effect of the intake of an oral probiotic containing of *W. cibaria* on patients with halitosis. The results of this clinical trial are meaningful since they supported the effect of *W. cibaria* on the improvement of halitosis.

The effects of probiotic agents on halitosis have been demonstrated using *Lactobacillus* strains in several probiotics studies (Keller et al., 2012; Suzuki et al., 2014; Penala et al., 2016). Lactic acid-producing bacteria, such as *Lactobacillus acidophilus* and *Lactobacillus casei*, have usually been chosen because of their inhibitory effect on anaerobic bacterial proliferation *via* the production of strong acids. However, this strong acid can be neutralized by the buffering function of saliva in the oral cavity of a healthy person and has the potential to induce dental caries (Babaahmady et al., 1998). In contrast, *W. cibaria* can prevent dental caries because it inhibits biofilm formation by *Streptococcus mutans* (Jang et al., 2016) and has a higher ecological pH than Lactobacilli strains (Kang et al., 2006). In light of these results, a randomized controlled clinical trial on the effect of *W. cibaria* CMU-containing probiotics on halitosis was conducted in this study. The total VSCs and the sum of H_2_S and CH_3_SH emitted from the mouth were measured. Usually, the concentration of VSCs is used as an indicator of halitosis severity (Rosenberg et al., 1991). The most common VSC is H_2_S from the back of the tongue (Washio et al., 2005), methyl mercaptans in the case of periodontal disease (Nakano et al., 2002b), and dimethyl sulfide, which originates extra-orally in the gut (Tangerman and Winkel, 2010). In our study, total VSC and sum of H_2_S and CH_3_SH showed similar patterns. Therefore, even if only one of the two aspects is measured, halitosis would be sufficiently evaluated in a clinical study. *W. cibaria* CMU-containing tablets significantly reduced the total VSC and the sum of H_2_S and CH_3_SH levels after week 8, and the number of *W. cibaria* in the mouth increased with a corresponding reduction in the severity of halitosis. These results are similar to those of previous studies where *W. cibaria* led to a reduction in the levels of H_2_S and CH_3_SH (Kang et al., 2006; Jang et al., 2016; Do et al., 2019). The results of our study also corroborate the findings from previous studies which showed that H_2_S and CH_3_SH concentrations decreased when participants gargled using mouthwash containing *W. cibaria* (Kang et al., 2006). Despite the differences in food type and treatment duration, both results showed an improvement in halitosis.

The decrease in total VSC and the sum of H_2_S and CH_3_SH over 8 weeks could be explained by the increase in the proportion of *W. cibaria*. Some gram-negative bacteria including *F. nucleatum*, *P. gingivalis*, *P. intermedia*, and *T. denticola* have been found to cause bad breath (Salako and Philip, 2011; Ouhara et al., 2015). These bacteria produce sulfur-containing compounds, such as H_2_S, by decomposing cysteine and methionine (Persson et al., 1990). The antibacterial effect of *W. cibaria* strains against representative oral bacteria, including *Aggregatibacter actinomycetemcomitans*, *F. nucleatum*, *P. gingivalis*, *Tannerella forsythia*, and *T. denticola*, has been reported in a previous study (Lim et al., 2018; Do et al., 2019). Among them, *F. nucleatum* serves as a bridge organism for other bacteria to engage in cohesion and coaggregation and can help them inhabit the oral cavity (Kolenbrander, 2000); *F. nucleatum* survives in the oral cavity because saliva cannot easily remove it (Jang et al., 2016). However, the proportion of *F. nucleatum* was found to decrease upon exposure to *W. cibaria* strains (Kang et al., 2006), and *W. cibaria* strains have been reported to strongly coaggregate with *F. nucleatum* and efficiently adhere to epithelial cells (Kang et al., 2005). In addition, *W. cibaria* produces higher levels of hydrogen peroxide, a representative antibacterial substance, than other *lactobacilli* (Jang et al., 2016). Hydrogen peroxide has been reported to alter the bacterial community in the oral cavity and inhibit the growth of *F. nucleatum* (Kang et al., 2006). The proportion of *F. nucleatum* could be significantly reduced in the oral cavity owing to the co-aggregation with *W. cibaria*, eliminating pathogenic bacteria thereby preventing VSC production (Kang et al., 2020).

Halitosis can be measured using an organoleptic method or gas chromatography analysis (Murata et al., 2002). In this study, the organoleptic method was not used to determined halitosis. The organoleptic method is practical and commonly used to measure bad breath; however, it has the limitation of less objectivity and low reproducibility since it is performed by directly sniffing the patient’s breath (Oho et al., 2001). Halitosis was measured by using the critical discrimination value as determined using gas chromatography based on a previous study that demonstrated the measurement of halitosis determined using gas chromatography to be correlated with the results of the organoleptic measurement (Murata et al., 2002).

The BBI scores had improved in the probiotics group than in the control group, similar to the results of another study (Lee et al., 2020). Bad breath is one of the common discomforts affecting many adults in modern society. Self-perceived bad breath is a factor that hinders the quality of life, owing to psychological discomfort, poor social ability, and social isolation (Bornstein et al., 2009; Kato et al., 2019). In the present study, it was shown that depression, oral health-related quality of life, and subjective oral health status had significantly improved in the probiotics group after 8 weeks; however, careful analysis is needed because we used subjective measures. For the safety evaluation of *W. cibaria* CMU-containing tablets, vital signs, hematology parameters, blood chemistry findings, and AEs were monitored. No significant problems were observed in the blood counts, liver function indicators, and renal function indicators, and no AEs were reported, suggesting that *W. cibaria* CMU-containing tablets could be safe for clinical application.

The purpose of this study was to evaluate whether *W. cibaria* CMU was settled in the test group, in which *W. cibaria* CMU-containing tablets were provided to the patients. The number of *W. cibaria* increased both in the test group and the control group in this study. However, a statistically significant difference between the two group, specifically increasing in the test group, was confirmed. For the measurement of *W. cibaria*, qPCR was quantified using *W. cibaria*-specific primer; however, whole genome sequencing was impossible. An increasing trend of *W. cibaria* in the control group may be explained by the fact that the patients who originally had *W. cibaria* showed it’s detected.

However, this study has a few limitations. First, the changes in the proportions of halitosis-inducing bacteria were not assessed. *W. cibaria* CMU has been known to inhibit the growth of VSC-producing bacteria by producing high amounts of hydrogen peroxide (Lim et al., 2018; Do et al., 2019). Many other studies based on *W. cibaria* CMU showed VSC reduction; however, this study did not show a statistically significant reduction in VSC in the probiotic group. Further studies are warranted to determine whether *W. cibaria* CMU significantly inhibits VSC-producing bacteria by assessing the amount of the bacteria. Second, despite randomization, it was difficult to find statistically significant differences between the groups because the concentrations of VSCs in the control and test groups were slightly different at baseline. Since total VSC and the sum of hydrogen sulfide and methylmercaptan were significantly higher in the control group from baseline, there seems to be a trend toward improvement in the control group over time. Further studies should be conducted with patients with similar baseline data to confirm the extensive clinical effects of *W. cibaria* on halitosis and mode of action of *W. cibaria* using next-generation sequencing technology.

The levels of *W. cibaria*, which, is known to led to a reduction in the levels of VSCs, is higher in patients with halitosis using *W. cibaria* CMU-containing tablets. The subjective degree of bad breath and psychological indicators were improved in patients with halitosis using *W. cibaria* CMU-containing tablets. *W. cibaria* CMU-containing tablets may be considered an adjunctive treatment for halitosis.

## Data availability statement

The original contributions presented in the study are included in the article/Supplementary material, further inquiries can be directed to the corresponding authors.

## Ethics statement

The studies involving human participants were reviewed and approved by Institutional Review Board of Seoul National University Dental Hospital (approval no. CRI19008). The patients/participants provided their written informed consent to participate in this study.

## Author contributions

H-sH and HY performed patient check-up, recorded clinical parameters, collected the samples, and analyzed the data. H-sH wrote the manuscript. Y-DC and SK supervised the research and revised the manuscript and corresponded. All authors contributed to the article and approved the submitted version.

## Funding

This work was supported by “Food Functionality Evaluation program” under the Ministry of Agriculture, Food and Rural Affairs and partly Korea Food Research Institute (G0190300-01).

## Conflict of interest

The authors declare that the research was conducted in the absence of any commercial or financial relationships that could be construed as a potential conflict of interest.

## Publisher’s note

All claims expressed in this article are solely those of the authors and do not necessarily represent those of their affiliated organizations, or those of the publisher, the editors and the reviewers. Any product that may be evaluated in this article, or claim that may be made by its manufacturer, is not guaranteed or endorsed by the publisher.
