# Supplementary material for: Improvement of halitosis by probiotic bacterium Weissella cibaria CMU: A randomized controlled trial
Source: Front Microbiol. 2023 Jan 17;14:1108762. doi: 10.3389/fmicb.2023.1108762 (PMC9886871; doi:10.3389/fmicb.2023.1108762)
Supplement: Supplementary file 1 [file Table_1.docx]

Supplementary Material

Improvement of halitosis by probiotic bacterium Weissella cibaria CMU: A randomized controlled trial

Hee-seung Han^1^, Haeji Yum^1^, Young-Dan Cho^1,†^, Sungtae Kim^1, †^

*** Correspondence:**

Sungtae Kim: kst72@snu.ac.kr

Young-Dan Cho: cacodm1@snu.ac.kr

Supplementary Material should be uploaded separately on submission. Please include any supplementary data, figures and/or tables.

Supplementary material is not typeset so please ensure that all information is clearly presented, the appropriate caption is included in the file and not in the manuscript, and that the style conforms to the rest of the article.

# Supplementary Tables

**Supplementary Table 1. BBI scores at 4 and 8 weeks**

| Week | Placebo | | | Probiotic | | | *p*-value^†^ |
| --- | --- | --- | --- | --- | --- | --- | --- |
| Week 4 | 2.2 | ± | 0.1 | 2.0 | ± | 0.1 | 0.138 |
| Week 8 | 2.3 | ± | 0.1 | 1.9 | ± | 0.1 | 0.006 |

Values are LSmean ± SE (all such values)
^†^ Student’s *t*-test was used to compare differences between the groups

**Supplementary Table 2. Social and psychological health biomarker measured at baseline, 4, and 8 weeks**

| Variables | Placebo | | | | Probiotic | | | | *p*-value ^†^ | | *p*-value ^‡^ | |
| --- | --- | --- | --- | --- | --- | --- | --- | --- | --- | --- | --- | --- |
| Depression | |  |  |  |  |  |  |  | |  | |  |
| Week 0 | | 13.1 | ± | 1.4 | 13.9 | ± | 1.3 | 0.740 | |  | |  |
| Week 8 | | 12.7 | ± | 1.4 | 12.7 | ± | 1.3 | 0.740 | | 0.345 | |  |
| *p*-value ^§^ | | 0.504 | | | 0.049 | | |  | |  | |  |
| Self-esteem | |  |  |  |  |  |  |  | |  | |  |
| Week 0 | | 39.5 | ± | 1.3 | 39.1 | ± | 1.3 | 0.706 | |  | |  |
| Week 8 | | 39.9 | ± | 1.3 | 38.9 | ± | 1.3 | 0.355 | | 0.473 | |  |
| *p*-value ^§^ | | 0.563 | | | 0.661 | | |  | |  | |  |
| Oral health-related quality of life | | | | |  |  |  |  | |  | |  |
| Week 0 | | 57.9 | ± | 1.9 | 57.2 | ± | 1.8 | 0.686 | |  | |  |
| Week 8 | | 59.1 | ± | 1.9 | 60.1 | ± | 1.8 | 0.562 | | 0.166 | |  |
| *p*-value ^§^ | | 0.139 | | | 0.001 | | |  | |  | |  |
| Subjective oral health status | | | | | | | |  | |  | |  |
| Week 0 | | 26.0 | ± | 1.4 | 26.2 | ± | 1.4 | 0.928 | |  | |  |
| Week 8 | | 25.3 | ± | 1.4 | 24.4 | ± | 1.4 | 0.291 | | 0.208 | |  |
| *p*-value ^§^ | | 0.336 | | | 0.007 | | |  | |  | |  |

Values are LSmean ± SE (all such values)

^†^ Student’s *t*-test was used to compare differences between the groups

^‡^ Linear mixed-effect model adjusted with total volatile sulfur compound, alcohol drinker and alcohol amount at baseline was used to analyze the effects of group*week

^§^ Linear mixed-effect model adjusted with total volatile sulfur compound, alcohol drinker and alcohol amount at baseline was used to analyze the difference within each group

**Supplementary Table 3. Vital signs at baseline, 4, and 8 weeks (unit: mmHg)**

| Variables | Placebo | | | Probiotic | | | *p*-value^†^ | | |
| --- | --- | --- | --- | --- | --- | --- | --- | --- | --- |
|  |  |  |  |  |  |  | Group | Week | Group*week |
| SBP |  |  |  |  |  |  |  |  |  |
| Week 0 | 119.5 | ± | 1.8 | 119.6 | ± | 1.8 |  |  |  |
| Week 4 | 117.6 | ± | 1.8 | 119.5 | ± | 1.8 |  |  |  |
| Week 8 | 118.5 | ± | 1.8 | 116.5 | ± | 1.8 | 0.994 | 0.148 | 0.163 |
| *p*-value^‡^ | 0.505 | | | 0.037 | | |  |  |  |
| DBP |  |  |  |  |  |  |  |  |  |
| Week 0 | 79.0 | ± | 1.3 | 79.5 | ± | 1.3 |  |  |  |
| Week 4 | 77.7 | ± | 1.3 | 79.0 | ± | 1.3 |  |  |  |
| Week 8 | 78.7 | ± | 1.3 | 76.3 | ± | 1.3 | 0.877 | 0.115 | 0.062 |
| *p*-value^‡^ | 0.796 | | | 0.008 | | |  |  |  |

Values are LSmean ± SE (all such values). SBP, systolic blood pressure; DBP, diastolic blood pressure

^†^ Linear mixed-effect model was used to analyze the effects of group, week and group*week

^‡^ Linear mixed-effect model was used to analyze the difference within each group

**Supplementary Table 4. Hematology at baseline and 8 weeks**

| Variables | Placebo | | | | | | Probiotic | | | | | | *p*-value ^†^ | | | | | |
| --- | --- | --- | --- | --- | --- | --- | --- | --- | --- | --- | --- | --- | --- | --- | --- | --- | --- | --- |
|  |  |  |  |  |  |  |  |  |  |  |  |  | Group | | Week | | Group*week | |
| WBC (10^3^/μL) |  | |  | |  | |  | |  | |  | |  | |  | |  | |
| Week 0 | 5.2 | | ± | | 0.2 | | 5.4 | | ± | | 0.2 | |  | |  | |  | |
| Week 8 | 5.2 | | ± | | 0.2 | | 5.7 | | ± | | 0.2 | | 0.185 | | 0.235 | | 0.392 | |
| *p*-value ^‡^ | 0.812 | | | | | | 0.152 | | | | | |  | |  | |  | |
| RBC (10^6^/μL) |  | |  | |  | |  | |  | |  | |  | |  | |  | |
| Week 0 | 4.61 | | ± | | 0.06 | | 4.58 | | ± | | 0.06 | |  | |  | |  | |
| Week 8 | 4.60 | | ± | | 0.06 | | 4.56 | | ± | | 0.06 | | 0.615 | | 0.294 | | 0.562 | |
| *p*-value ^‡^ | 0.738 | | | | | | 0.253 | | | | | |  | |  | |  | |
| Hb (g/dL) |  | |  | |  | |  | |  | |  | |  | |  | |  | |
| Week 0 | 14.0 | | ± | | 0.2 | | 13.8 | | ± | | 0.2 | |  | |  | |  | |
| Week 8 | 13.9 | | ± | | 0.2 | | 13.7 | | ± | | 0.2 | | 0.416 | | 0.102 | | 0.848 | |
| *p*-value ^‡^ | 0.302 | | | | | | 0.198 | | | | | |  | |  | |  | |
| Hct (%) |  | |  | |  | |  | |  | |  | |  | |  | |  | |
| Week 0 | 41.6 | | ± | | 0.4 | | 41.2 | | ± | | 0.5 | |  | |  | |  | |
| Week 8 | 41.4 | | ± | | 0.4 | | 40.8 | | ± | | 0.5 | | 0.389 | | 0.033 | | 0.431 | |
| *p*-value ^‡^ | 0.332 | | | | | | 0.041 | | | | | |  | |  | |  | |
| MCV (fL) |  | |  | |  | |  | |  | |  | |  | |  | |  | |
| Week 0 | 90.4 | | ± | | 0.8 | | 90.1 | | ± | | 0.8 | |  | |  | |  | |
| Week 8 | 90.2 | | ± | | 0.8 | | 89.8 | | ± | | 0.8 | | 0.740 | | 0.034 | | 0.827 | |
| *p*-value ^‡^ | 0.173 | | | | | | 0.099 | | | | | |  | |  | |  | |
| MCH (pg) |  | |  | |  | |  | |  | |  | |  | |  | |  | |
| Week 0 | 30.3 | | ± | | 0.3 | | 30.1 | | ± | | 0.3 | |  | |  | |  | |
| Week 8 | 30.2 | | ± | | 0.3 | | 30.1 | | ± | | 0.3 | | 0.734 | | 0.248 | | 0.415 | |
| *p*-value ^‡^ | 0.163 | | | | | | 0.810 | | | | | |  | |  | |  | |
| MCHC (g/dL) |  | |  | |  | |  | |  | |  | |  | |  | |  | |
| Week 0 | 33.5 | | ± | | 0.2 | | 33.4 | | ± | | 0.2 | |  | |  | |  | |
| Week 8 | 33.5 | | ± | | 0.2 | | 33.5 | | ± | | 0.2 | | 0.763 | | 0.616 | | 0.356 | |
| *p*-value ^‡^ | 0.764 | | | | | | 0.317 | | | | | |  | |  | |  | |
| PLT (10^3^/μL) |  | |  | |  | |  | |  | |  | |  | |  | |  | |
| Week 0 | 243.5 | | ± | | 7.6 | | 264.4 | | ± | | 7.7 | |  | |  | |  | |
| Week 8 | 239.3 | | ± | | 7.6 | | 261.4 | | ± | | 7.7 | | 0.043 | | 0.157 | | 0.822 | |
| *p*-value ^‡^ | 0.244 | | | | | | 0.401 | | | | | |  | |  | |  | |
| Neutrophil (%) | |  | |  | |  | |  | |  | |  | |  | |  | |  |
| Week 0 | | 55.8 | | ± | | 1.2 | | 54.8 | | ± | | 1.2 | |  | |  | |  |
| Week 8 | | 56.0 | | ± | | 1.2 | | 56.1 | | ± | | 1.2 | | 0.754 | | 0.335 | | 0.428 |
| *p*-value ^‡^ | | 0.902 | | | | | | 0.218 | | | | | |  | |  | |  |
| Lymphocyte (%) | |  | |  | |  | |  | |  | |  | |  | |  | |  |
| Week 0 | | 33.7 | | ± | | 1.1 | | 34.7 | | ± | | 1.1 | |  | |  | |  |
| Week 8 | | 33.7 | | ± | | 1.1 | | 33.1 | | ± | | 1.1 | | 0.925 | | 0.212 | | 0.223 |
| *p*-value ^‡^ | | 0.983 | | | | | | 0.084 | | | | | |  | |  | |  |
| Monocyte (%) | |  | |  | |  | |  | |  | |  | |  | |  | |  |
| Week 0 | | 7.8 | | ± | | 0.3 | | 7.5 | | ± | | 0.3 | |  | |  | |  |
| Week 8 | | 7.6 | | ± | | 0.3 | | 7.6 | | ± | | 0.3 | | 0.613 | | 0.804 | | 0.430 |
| *p*-value ^‡^ | | 0.461 | | | | | | 0.703 | | | | | |  | |  | |  |
| Eosinophil (%) | |  | |  | |  | |  | |  | |  | |  | |  | |  |
| Week 0 | | 2.3 | | ± | | 0.3 | | 2.7 | | ± | | 0.3 | |  | |  | |  |
| Week 8 | | 2.3 | | ± | | 0.3 | | 2.8 | | ± | | 0.3 | | 0.207 | | 0.510 | | 0.572 |
| *p*-value ^‡^ | | 0.946 | | | | | | 0.390 | | | | | |  | |  | |  |
| Basophil (%) | |  | |  | |  | |  | |  | |  | |  | |  | |  |
| Week 0 | | 0.35 | | ± | | 0.04 | | 0.41 | | ± | | 0.04 | |  | |  | |  |
| Week 8 | | 0.42 | | ± | | 0.04 | | 0.41 | | ± | | 0.04 | | 0.618 | | 0.124 | | 0.101 |
| *p*-value ^‡^ | | 0.025 | | | | | | 0.943 | | | | | |  | |  | |  |

Values are LSmean ± SE (all such values). WBC, white blood cell; RBC, red blood cell; Hb, hemoglobin; Hct, hematocrit; MCV, mean corpuscular volume; MCH, mean corpuscular hemoglobin; MCHC, mean corpuscular hemoglobin concentration; PLT, platelet.

^†^ Linear mixed-effect model was used to analyze the effects of group, week and group*week.

^‡^ Linear mixed-effect model was used to analyze the difference within each group.

**Supplementary Table 5. Blood chemistry at baseline and 8 weeks**

| Variables | Placebo | | | Probiotic | | | *p*-value ^†^ | | |
| --- | --- | --- | --- | --- | --- | --- | --- | --- | --- |
|  |  |  |  |  |  |  | Group | Week | Group*week |
| Glucose (mg/dL) |  |  |  |  |  |  |  |  |  |
| Week 0 | 95.5 | ± | 1.8 | 101.2 | ± | 1.8 |  |  |  |
| Week 8 | 97.3 | ± | 1.8 | 103.1 | ± | 1.8 | 0.020 | 0.006 | 0.934 |
| *p*-value ^‡^ | 0.054 | | | 0.044 | | |  |  |  |
| AST (IU/L) |  |  |  |  |  |  |  |  |  |
| Week 0 | 23.3 | ± | 1.3 | 24.7 | ± | 1.3 |  |  |  |
| Week 8 | 26.0 | ± | 1.3 | 24.5 | ± | 1.3 | 0.975 | 0.155 | 0.083 |
| *p*-value ^‡^ | 0.026 | | | 0.825 | | |  |  |  |
| ALT (IU/L) |  |  |  |  |  |  |  |  |  |
| Week 0 | 22.5 | ± | 2.5 | 23.6 | ± | 2.5 |  |  |  |
| Week 8 | 27.5 | ± | 2.5 | 24.1 | ± | 2.5 | 0.688 | 0.174 | 0.267 |
| *p*-value ^‡^ | 0.080 | | | 0.858 | | |  |  |  |
| Total protein (g/dL) |  |  |  |  |  |  |  |  |  |
| Week 0 | 7.3 | ± | 0.0 | 7.4 | ± | 0.1 |  |  |  |
| Week 8 | 7.3 | ± | 0.0 | 7.4 | ± | 0.1 | 0.117 | 0.078 | 0.987 |
| *p* -value ^‡^ | 0.203 | | | 0.217 | | |  |  |  |
| TC (mg/dL) |  |  |  |  |  |  |  |  |  |
| Week 0 | 201.5 | ± | 4.6 | 200.0 | ± | 4.6 |  |  |  |
| Week 8 | 196.4 | ± | 4.6 | 196.9 | ± | 4.6 | 0.934 | 0.159 | 0.728 |
| *p*-value ^‡^ | 0.212 | | | 0.453 | | |  |  |  |
| BUN (mg/dL) |  |  |  |  |  |  |  |  |  |
| Week 0 | 14.2 | ± | 0.5 | 13.9 | ± | 0.5 |  |  |  |
| Week 8 | 13.5 | ± | 0.5 | 14.4 | ± | 0.5 | 0.611 | 0.655 | 0.065 |
| *p*-value ^‡^ | 0.103 | | | 0.320 | | |  |  |  |
| Creatinine (mg/dL) |  |  |  |  |  |  |  |  |  |
| Week 0 | 0.85 | ± | 0.02 | 0.82 | ± | 0.02 |  |  |  |
| Week 8 | 0.83 | ± | 0.02 | 0.83 | ± | 0.02 | 0.547 | 0.218 | 0.061 |
| *p*-value ^‡^ | 0.028 | | | 0.646 | | |  |  |  |
| Uric acid (mg/dL) |  |  |  |  |  |  |  |  |  |
| Week 0 | 4.9 | ± | 0.2 | 4.8 | ± | 0.2 |  |  |  |
| Week 8 | 4.7 | ± | 0.2 | 4.6 | ± | 0.2 | 0.719 | 0.005 | 0.565 |
| *p*-value ^‡^ | 0.106 | | | 0.017 | | |  |  |  |
| Calcium (mg/dL) |  |  |  |  |  |  |  |  |  |
| Week 0 | 9.1 | ± | 0.0 | 9.2 | ± | 0.0 |  |  |  |
| Week 8 | 9.0 | ± | 0.0 | 9.1 | ± | 0.0 | 0.260 | 0.000 | 0.668 |
| *p*-value ^‡^ | 0.024 | | | 0.005 | | |  |  |  |
| Phosphorus (mg/dL) |  |  |  |  |  |  |  |  |  |
| Week 0 | 3.4 | ± | 0.1 | 3.4 | ± | 0.1 |  |  |  |
| Week 8 | 3.3 | ± | 0.1 | 3.4 | ± | 0.1 | 0.656 | 0.529 | 0.060 |
| *p*-value ^‡^ | 0.075 | | | 0.374 | | |  |  |  |

Values are LSmean ± SE (all such values). AST, aspartate aminotransferase; ALT, alanine transaminase; TC, total cholesterol; BUN, blood urea nitrogen.

^†^Linear mixed-effect model was used to analyze the effects of group, week and group*week.

^‡^Linear mixed-effect model was used to analyze the difference within each group.

**Supplementary Table 6. Adverse event**

| Variables | Placebo | Probiotic | *p*-value ^†^ |
| --- | --- | --- | --- |
| Occurrence |  |  |  |
| Adverse events (AE) | 8 / 9 | 3 / 4 | 0.117 |
| Serious adverse events (SAE) | 0 / 0 | 0 / 0 | - |
| Type |  |  |  |
| Cold | 1 / 1 | 1 / 2 | 1.000 |
| Arthritis | 2 / 2 | 0 / 0 | 0.495 |
| Headache | 1 / 1 | 0 / 0 | 1.000 |
| Mild fever | 2 / 2 | 0 / 0 | 0.495 |
| Trauma of instep | 0 / 0 | 1 / 1 | 0.495 |
| Ankle arthritis | 1 / 1 | 0 / 0 | 1.000 |
| Pain at finger joint | 1 / 1 | 0 / 0 | 1.000 |
| Tremor in fingers and toes | 1 / 1 | 0 / 0 | 1.000 |
| Vaginitis | 0 / 0 | 1 / 1 | 0.495 |
| Level of symptoms |  |  |  |
| Mild | 8 / 9 | 3 / 4 | 0.117 |
| Moderate | 0 / 0 | 0 / 0 | - |
| Severe | 0 / 0 | 0 / 0 | - |
| Relevance to test food |  |  |  |
| Clearly related | 0 / 0 | 0 / 0 | - |
| Though to be related | 0 / 0 | 0 / 0 | - |
| Likely to be related | 0 / 0 | 0 / 0 | - |
| Not thought to be related | 3 / 4 | 0 / 0 | 0.242 |
| Clearly not related | 5 / 5 | 3 / 4 | 0.714 |
| Unknown | 0 / 0 | 0 / 0 | - |

Values are number of subjects / number of cases.

^†^Chi-square test or Fisher’s exact test was used to compare the difference between the groups.
